# Supplementary figures and images for: Recombinant expression in E. coli of human FGFR2 with its transmembrane and extracellular domains
Source: PeerJ. 2017 Jun 29;5:e3512. doi: 10.7717/peerj.3512 (PMC5493969; doi:10.7717/peerj.3512)

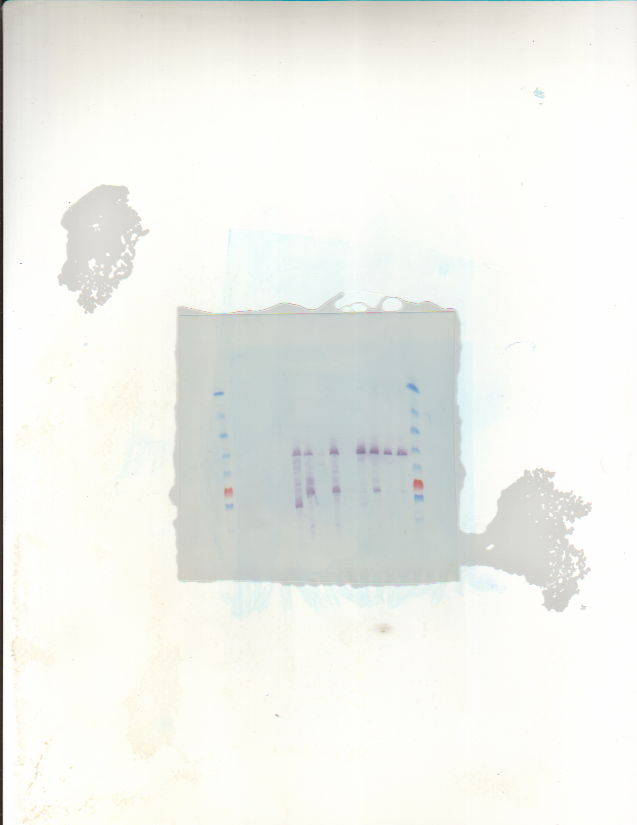


1 2 3 4 5 6 7 8 9 10 11 12 13 14

Supplement: Supplemental Information 1 — Figures 2–4, 6. [file peerj-05-3512-s001.zip › Fig2 western blot.docx]

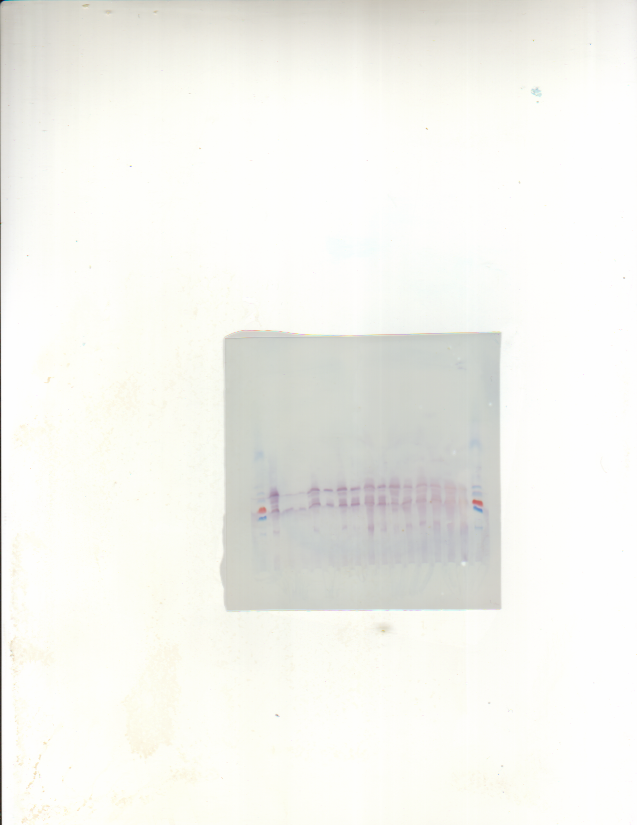


1 2 3 4 5 6 7 8 9 10 11 12 13

Supplement: Supplemental Information 1 — Figures 2–4, 6. [file peerj-05-3512-s001.zip › Fig3 western blot.docx]

*
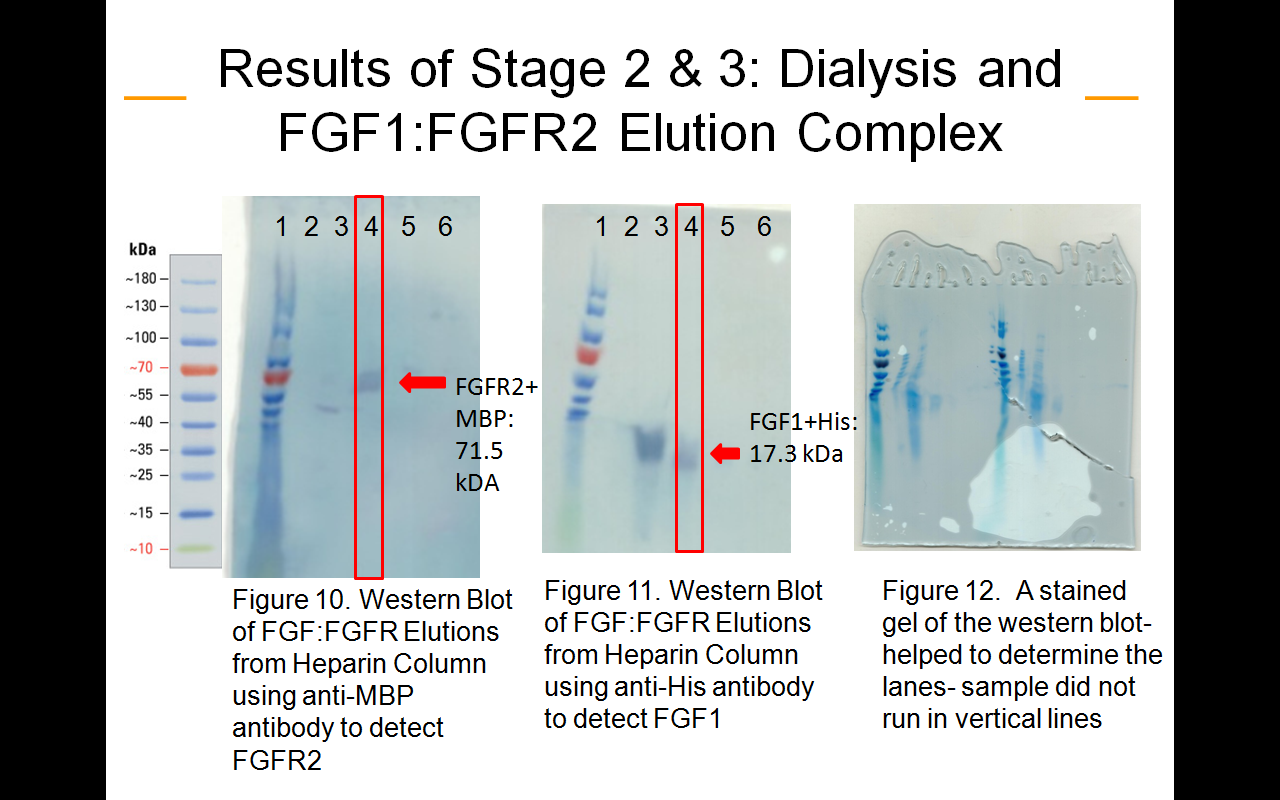
*

Supplement: Supplemental Information 1 — Figures 2–4, 6. [file peerj-05-3512-s001.zip › Fig4 western blot.docx]

1 2 3 4 5 6 7 8


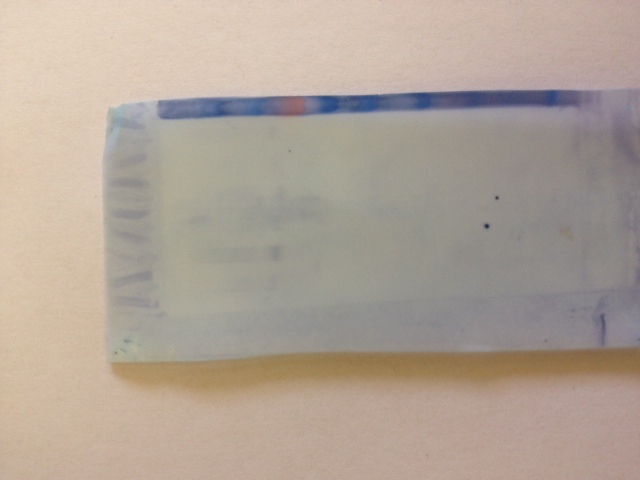


Fig. 6 in the paper comes from Lanes 1 (ladder) and 7.

Supplement: Supplemental Information 1 — Figures 2–4, 6. [file peerj-05-3512-s001.zip › Fig6 western blot.docx]
